# Cyclopeptides from *Amanita exitialis*

**DOI:** 10.1007/s13659-011-0013-9

**Published:** 2011-09-12

**Authors:** Jing-Hua Xue, Ping Wu, Yu-Lang Chi, Liang-Xiong Xu, Xiao-Yi Wei

**Affiliations:** Key Laboratory of Plant Resources Conservation and Sustainable Utilization, South China Botanical Garden, Chinese Academy of Sciences, Xingke Road 723, Tianhe District, Guangzhou, 510650 China

**Keywords:** *Amanita*, *Amanita exitialis*, cyclopeptides, amanexitide

## Abstract

A new cyclic nonapeptide, amanexitide (**1**), along with the known cyclopeptides, *α*- and *β*-amanitins, was isolated from the fruiting bodies of *Amanita exitialis*, a newly described poisonous mushroom endemic to Guangzhou, Guangdong Province, China. Its amino acid composition and absolute configuration were determined by chemical degradation and derivatization followed by chiral GC analysis. Its amino acid sequence was elucidated by extensive analysis of ESIMS/MS and FTICRMS data. The occurrence of **1** in this mushroom is interesting because it has a structure closely related to antamanide, a cyclic decapeptide with antidote activity against amatoxins and phallotoxins, occurring in *A. phalloides*.

## Introduction

*Amanita* mushrooms are well known for the toxic properties of some species.[1] Their toxins are demonstrated to be cyclopeptides, including amatoxins, phallotoxins, and virotoxins, which all contain a sulfur-linked tryptophan unit and some unusual hydroxylated amino acids.[1] Among these toxins amatoxins are considered to be the only group responsible for fatal mushroom poisoning.[2] The mechanism of amatoxins is the inhibition of transcription from DNA to mRNA by the blockade of nuclear RNA polymerase II.[2] Interestingly, a cyclic decapeptide, antamanide, acting as a potential anti-toxin against the effects of phallotoxins and amatoxins has been also isolated from an *Amanita* mushroom, the death cap *A. phalloides*.[3,4]

*Amanita exitialis*, endemic to Guangzhou, Guangdong Province, China, is a new *Amanita* species originally described in 2001.[5] This mushroom annually comes out at the beginning of March and disappears at the end of April in the north suburbs of Guangzhou. It has caused the death of more than 20 persons by the ingestion of it due to confusion with some wild edible mushrooms growing in the same area since 2000 when the first incidence of its poisoning was reported.[6] A previous HPLC analysis detected amatoxins and phallotoxins from the fruiting bodies of this mushroom.[7] For better understanding of its metabolites, we carried out a chemical investigation on this mushroom. We previously reported the isolation of a new purine nucleoside, $N^2$-(1-methoxycarbonylethyl)guanosine, along with *β*-carboline and russulaceramide.[8] Herein, we report the isolation and structure elucidation of a new cyclic nonapeptide, amanexitide (**1**), along with the known amatoxins, *α*- and *β*-amanitins from this species.

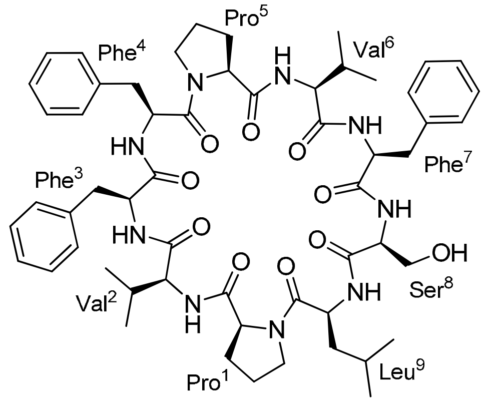

**1**

## Results and Discussion

The fresh fruiting bodies of *A. exitialis* were extracted with 95% EtOH at room temperature. The EtOH extract was successively partitioned with petroleum ether and EtOAc. The EtOAc-soluble fraction was separated by silica gel column chromatography to give amanexitide (**1**) as colorless needles. The water-soluble fraction was separated by Diaion HP-20 and silica gel column chromatography followed by HPLC to provide *α*-amanitin and *β*-amanitin,[9] which were identified by interpretation of their spectroscopic data (ESIMS, $^1$H and $^{13}$C NMR) as well as by comparison with reported data.

*To whom correspondence should be addressed. E-mail: wxy@scbg.ac.cn

Compound **1** was a cyclic peptide as characterized by acid hydrolysis and ninhydrin reaction on TLC plates using the method described by Zhou and Tan.[10] To determine the amino acid composition and absolute configuration, the compound was hydrolyzed with 6 N HCl containing 4% thioglycolic acid and the resultant amino acids were converted into the 2-propyl esters of their trifluoroacetylated derivatives.[11] The mixture of the prepared amino acid derivatives was subjected to GC-MS analysis with a chiral capillary column and the results showed that the cyclopeptide consisted of phenylalanine (Phe), proline (Pro), valine (Val), leucine (Leu), and serine (Ser), and these amino acids all had the L configuration.

The molecular weight 1033 for amanexitide (**1**) was deduced from the quasi-molecular ions at *m/z* 1072 $[M + K]^+$, 1056 $[M + Na]^+$, and 1034 $[M + H]^+$ in the positive ESIMS and *m/z* 1068 $[M + Cl]^-$ and 1032 $[M - H]^-$ in the negative ESIMS. Its molecular formula $C_{56}H_{75}N_9O_{10}$ was assigned on the basis of the quasi-molecular ion at *m/z* 1034.5715 $[M + H]^+$ (calcd for $C_{56}H_{75}N_9O_{10}$, 1034.5715) in the HRESIMS. This in combination with the results of GC-MS analysis deduced that compound **1** was comprised of three Phe, two Pro, two Val, one Leu, and one Ser per molecule. When this cyclopeptide was subjected to NMR measurements using pyridine-$d_5$ and $CDCl_3$ as solvents, it provided sets of broad proton and carbon signals due to the existence of multiple conformers in the both solvents. The measurements in other solvents were prevented from by the poor solubility. Sequence analysis of amanexitide using NMR technique failed. However, it has been demonstrated that proline-containing cyclopeptides can be readily sequenced by mass spectrometry because they undergo selective ring cleavage at the prolyl-peptidyl amide bond due to the more basic nature of the proline nitrogen relative to the other peptide bond nitrogen atoms, leading to a linear peptide C-ended by an acylium ion ($b_n$), which undergoes further fragmentation generating series of acylium ions.[12] Therefore, sequence analysis of this proline-containing cyclopeptide was conducted by the tandem electrospray-ionization mass spectrometry (ESIMS) technique.

The protonated molecular ion $[M + H]^+$ of **1** at *m/z* 1034.5 was subjected to collision-activated dissociation (CAD) experiment. In the product-ion spectrum (Figure 1), two Pro-directed fragmentation pathways (Figure 2) were observed. One started from ring opening at the Pro-Leu amide bond and a series of adjacent acylium ions ($b_{nPL}$) at *m/z* 921, 834, 687, 588, 491, 344, and 197 could be seen. The sequential loss of amino acid residues from the C-terminus to the N-terminus of the linearized amanexitide (**1**) was that of Leu, Ser, Phe, Val, Pro, Phe, and Phe yielding the N-terminal dipeptide ion $[HPro\text{-}Val]^+$ (Figure 2A). This pathway was also recognized by the series of adjacent immonium ions ($a_{nPL}$) at *m/z* 1006, 806, 659, 560, 463, 316, and 169 due to the loss of a molecule of carbon monoxide (CO) from the $[M + H]^+$ ion and the above $b_{7PL}$–$b_{2PL}$ ions. Only two ions at *m/z* 1016 and 903 corresponding to the loss of a molecule of water ($H_2O$) from the $[M + H]^+$ and the $b_{8PL}$ ions were observed while those from other $b_{nPL}$ fragments were not observed. Another fragmentation pathway was initiated by the ring cleavage at Pro-Phe amide bond. The series of $b_{nPF}$ ions at *m/z* 887, 740, 544, 431, 344, and 197 suggested the successive loss of Phe, Phe, Val-Pro, Leu, Ser, and Phe yielding $[HPro\text{-}Val]^+$ (Figure 2B), the same N-terminal dipeptide ion as that in the first pathway. The series of $a_{nPF}$ ions due to the loss of a CO from these $b_{nPF}$ fragments was observed at *m/z* 859, 712, 516, 403, 316, and 169. A short series of ions observed at *m/z* 869, 722, 526, and 413 was due to the loss of a $H_2O$ from the first four $b_{nPF}$ ($b_{8PF}$, $b_{7PF}$, $b_{5PF}$, and $b_{4PF}$) fragments. These data suggested the sequence $[H\text{-}Pro^1\text{-}Val^2\text{-}Phe^3\text{-}Phe^4\text{-}Pro^5\text{-}Val^6\text{-}Phe^7\text{-}Ser^8\text{-}Leu^9]^+$ for the linearized peptide ion formed from amanexitide (**1**).

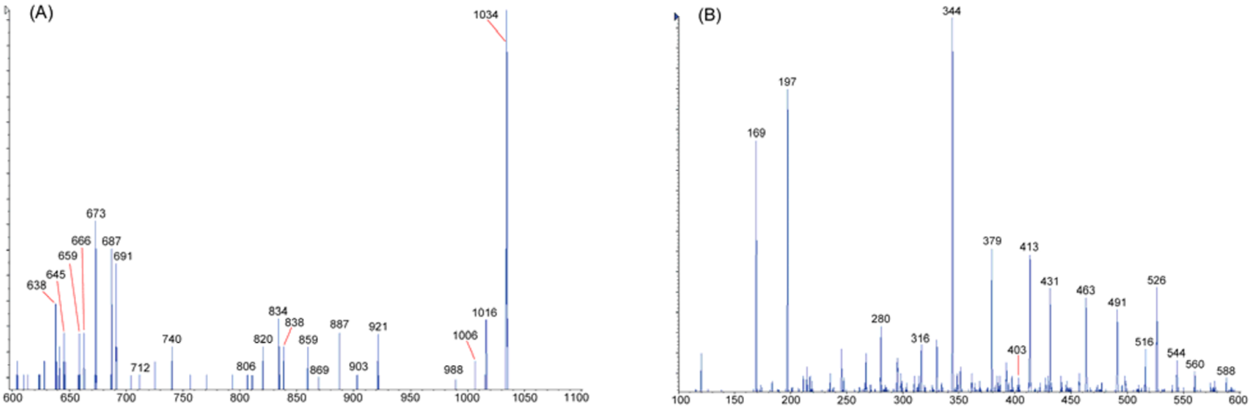

**Figure 1.** Positive ion $ESIMS^2$ spectrum of $[M + H]^+$ ion at *m/z* 1034.5 for compound **1**: (A) *m/z* 600—1060; (B) *m/z* 100—600.

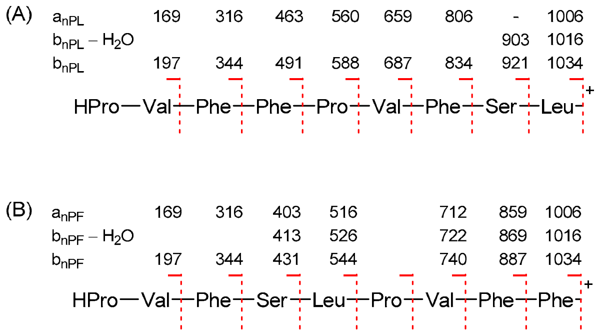

**Figure 2.** Two Pro-directed CAD fragmentation pathways of compound **1**: (A) Cleavage at the $Pro^1\text{-}Leu^9$ amide bond level; (B) cleavage at the $Pro^5\text{-}Phe^4$ amide bond level.

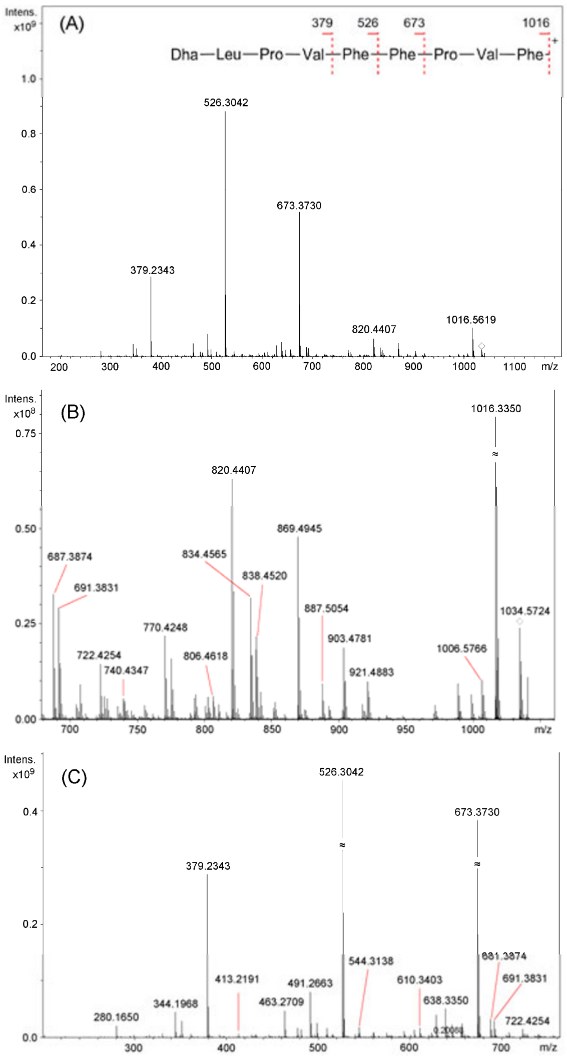

**Figure 3.** Positive FTICRMS/MS spectrum of $[M + H]^+$ at *m/z* 1034.5 for compound **1**: (A) The whole spectrum and the assignment of predominant ions, (B) *m/z* 680–1060, (C) *m/z* 200–760.

In order to confirm the above fragments, the protonated molecular ion $[M + H]^+$ of **1** at *m/z* 1034.5 was subjected to SORI-CID experiment on a Fourier Transform Ion Cyclotron Resonance Mass Spectrometry (FTICRMS). In the product-ion spectrum (Figure 3), three ions at *m/z* 673.3730, 526.3042, and 379.2343 were extremely abundant (Figure 3A). They apparently resulted from ring-opening at the Ser-Phe amide bond followed by the loss of a $H_2O$ from the serine residue to produce a fragment with 2,3-didehydroalanine (Dha) as the N-terminal residue at *m/z* 1016.5619, which underwent further fragmentation by sequential loss of Pro-Val-Phe, Phe, and Phe yielding the N-terminal tetrapeptide Dha-Leu-Pro-Val (Figure 3A). The most ions stated above for the two Pro-directed fragmentation pathways were also observed (Table 1) though some were weak, and their exact masses were in accord with the predicted values as shown in Table 1. Therefore, the structure of amanexitide was elucidated as shown. The geometry of the peptidic linkages at the proline residues was not determined due to the poor solubility restraining detailed NMR experiments.

Compound **1** was evaluated for cytotoxicity against various cancer cells (A549, LAC, HeLa, HepG2, HL-60, KB, GH3, and AT3)[13] and inhibition of the production of pro-inflammatory cytokines, TNFα and IL-6, in lipopolysaccharide (LPS)-stimulated J774A.1 macrophages,[14] but was found to be inactive in both assays. Nevertheless, it is interesting that the sequence of the first five amino acid residues, $Pro^1$-$Val^2$-$Phe^3$-$Phe^4$-$Pro^5$, in amanexitide (**1**) is the same as that in antamanide, a cyclic decapeptide previously isolated as an antidote for poisoning of amatoxins and phallotoxins from *A. phalloides*.[2, 4] This in combination with the isolation of $\alpha$- and $\beta$-amatoxins showed that *A. exitialis* may be close to *A. phalloides* in genetic relationship. In addition, compound **1**, having a structure related to antamanide, deserves further investigation of its antidote activity against amatoxins and phallotoxins.

## Experimental Section

**General Experimental Procedures.** The melting point was determined on a Yanagimoto Seisakusho MD-S2 micro-melting point apparatus and was uncorrected. Optical rotations were obtained on a Perkin-Elmer 341 polarimeter with MeOH as solvent. The $^1H$ and $^{13}C$ NMR spectra were recorded on a Bruker Avance-600 instrument. HRESIMS data were obtained on a Bruker Bio TOF IIIQ mass spectrometer. Preparative HPLC was performed with a Shimadzu LC-6A pump and a Shimadzu RID-10A refractive index detector using an XTerra prep MS $C_{18}$ column (10 $\mu$m, 300 × 19 mm). For column chromatography, silica gel 60 (100–200 mesh, Qingdao Marine Chemical Ltd., Qingdao, China) and Diaion HP-20 (200–600 $\mu$m, Mitsubishi Chemical Co., Tokyo, Japan) were used.

**Mushroom Material.** The fresh fruiting bodies of *A. exitialis* were collected at South China Botanical Garden, Chinese Academy of Sciences, Guangzhou, in March, 2006. The mushroom was authenticated by Prof. Tai-Hui Li, Guangdong Institute of Microbiology, Guangzhou.

**Extraction and Isolation.** The fresh fruiting bodies of *A. exitialis* (13.8 kg) were cut and crushed into a pulpy mass with an electric blender. EtOH (95%, 15 L) was added and the mixture was allowed to stand at room temperature for 24 h. The thick suspension was then filtered and the residue was further extracted two times with 95% EtOH (15 L each). The filtrates of all three extractions were combined and evaporated. The resulting brown syrup (440 g) was suspended in 1000 mL of water. The suspension was successively partitioned with petroleum ether (3 × 1000 mL) and EtOAc (4 × 1000 mL). The EtOAc layer was evaporated under vacuum to yield an EtOAc-soluble fraction (6.1 g). This fraction was subjected to silica gel CC eluted with a gradient of $CHCl_3$-MeOH (100:0–80:20) to give seven fractions (E1–E7). Fraction E4, obtained on elution with $CHCl_3$-MeOH (90:10), was purified by recrystallization in MeOH to give amanexitide (**1**) as colorless needles (34 mg). The aqueous layer was concentrated in vacuum to remove the residual EtOAc and then filtered. A portion (150 mL) of the filtrate (750 mL) was subjected to passage over a Diaion HP-20 column, sequentially eluted with $H_2O$, 20%

EtOH, and 95% EtOH. The fractions obtained by elution with 20% EtOH and 95% EtOH was combined and concentrated to provide a residue (8.5 g). This residue was subjected to a silica gel column eluted with $CHCl_3$-MeOH mixtures of increasing polarity (9:1 to 5:5) to give six fractions (W1–W6). Fraction W3 (1.61 g), obtained by elution with $CHCl_3$-MeOH (7:3), was separated by preparative HPLC using 12% MeOH to afford $\alpha$-amanitin (28 mg). Fraction W4 (1.30 g), obtained by elution with $CHCl_3$-MeOH (6:4), was also separated by preparative HPLC using 12% MeOH to afford $\beta$-amanitin (8 mg).

**Table 1. Significant FTICRMS ions of compound 1.**

| fragment ion | composition | found *m/z* | intensity | calcd *m/z* | error (ppm) |
|---|---|---|---|---|---|
| M + H | $C_{56}H_{76}N_9O_{10}$ | 1034.5724 | 24708093 | 1034.5715 | + 0.87 |
| M + H – $H_2O$ | $C_{56}H_{74}N_9O_9$ | 1016.5619 | 104039817 | 1016.5610 | + 0.96 |
| M + H – CO | $C_{55}H_{76}N_9O_9$ | 1006.5766 | 10600576 | 1006.5766 | + 0.03 |
| M + H – $H_2O$ – CO | $C_{55}H_{74}N_9O_8$ | 988.5660 | 9870132 | 988.5660 | – 0.04 |
| $b_{8PL}$ | $C_{50}H_{65}N_8O_9$ | 921.4883 | 10234173 | 921.4875 | + 0.95 |
| $b_{8PL}$ – $H_2O$ | $C_{50}H_{63}N_8O_8$ | 903.4781 | 19280059 | 903.4769 | + 1.33 |
| $b_{8PF}$ | $C_{47}H_{67}N_8O_9$ | 887.5054 | 9484268 | 887.5031 | + 2.63 |
| $b_{8PF}$ – $H_2O$ | $C_{47}H_{65}N_8O_8$ | 869.4945 | 49542104 | 869.4925 | + 2.27 |
| $a_{8PF}$ | $C_{46}H_{67}N_8O_8$ | 859.5096 | 1328446 | 859.5082 | + 1.58 |
| M + H – $b_{2PL}$/$b_{2PF}$ | $C_{46}H_{60}N_7O_8$ | 838.4520 | 22105954 | 838.4503 | + 2.02 |
| $b_{7PL}$ | $C_{47}H_{60}N_7O_7$ | 834.4565 | 32694676 | 834.4554 | + 1.31 |
| M + H – $b_{2PL}$/$b_{2PF}$ – $H_2O$ | $C_{46}H_{58}N_7O_7$ | 820.4407 | 65171127 | 820.4398 | + 1.13 |
| M + H – $b_{2PL}$/$b_{2PF}$ – CO | $C_{45}H_{60}N_7O_7$ | 810.4574 | 4027286 | 810.4554 | + 2.44 |
| $a_{7PL}$ | $C_{46}H_{60}N_7O_6$ | 806.4618 | 6264517 | 806.4605 | + 1.56 |
| $b_{7SF}$ – $H_2O$ | $C_{42}H_{56}N_7O_7$ | 770.4248 | 22546114 | 770.4241 | + 0.90 |
| $b_{7PF}$ | $C_{38}H_{58}N_7O_8$ | 740.4347 | 5041794 | 740.4347 | + 0.03 |
| $b_{7PF}$ – $H_2O$ | $C_{38}H_{56}N_7O_7$ | 722.4254 | 14513248 | 722.4241 | + 1.81 |
| $a_{7PF}$ | $C_{37}H_{58}N_7O_7$ | 712.4408 | 690224 | 712.4398 | + 1.47 |
| $b_{6SF}$ | $C_{37}H_{51}N_6O_7$ | 691.3831 | 30506526 | 691.3819 | + 1.71 |
| $b_{6PL}$ | $C_{38}H_{51}N_6O_6$ | 687.3874 | 33152698 | 687.3870 | + 0.52 |
| $b_{6SF}$ – $H_2O$ | $C_{37}H_{49}N_6O_6$ | 673.3730 | 517959322 | 673.3714 | + 2.41 |
| $a_{6SF}$ | $C_{36}H_{51}N_6O_6$ | 663.3877 | 2921004 | 663.3870 | + 1.03 |
| $a_{6PL}$ | $C_{37}H_{51}N_6O_5$ | 659.3936 | 4814472 | 659.3921 | + 2.35 |
| M + H – $b_{4SF}$ | $C_{37}H_{44}N_5O_5$ | 638.3350 | 51474859 | 638.3342 | + 1.24 |
| M + H – $b_{4SF}$ – CO | $C_{36}H_{44}N_5O_4$ | 610.3403 | 16968414 | 610.3393 | + 1.62 |
| $b_{5PL}$ | $C_{33}H_{42}N_5O_5$ | 588.3195 | 2720318 | 588.3186 | + 1.53 |
| $b_{5PF}$ | $C_{28}H_{42}N_5O_6$ | 544.3138 | 18142810 | 544.3135 | + 0.57 |
| $b_{5PF}$ – $H_2O$ | $C_{28}H_{40}N_5O_5$ | 526.3042 | 872095946 | 526.3029 | + 2.30 |
| $a_{5PF}$ | $C_{27}H_{42}N_5O_5$ | 516.3196 | 4329125 | 516.3186 | + 1.96 |
| $a_{5SF}$ – $H_2O$ | $C_{27}H_{40}N_5O_4$ | 498.3086 | 25513729 | 498.3080 | + 1.04 |
| $b_{4PL}$ | $C_{28}H_{35}N_4O_4$ | 491.2663 | 80915682 | 491.2658 | + 0.92 |
| $a_{4PL}$ | $C_{27}H_{35}N_4O_3$ | 463.2709 | 46818520 | 463.2709 | + 0.00 |
| $b_{4PF}$ – $H_2O$ | $C_{22}H_{29}N_4O_4$ | 413.2191 | 5625672 | 413.2189 | + 0.53 |
| $b_{4SF}$ – $H_2O$ | $C_{19}H_{31}N_4O_4$ | 379.2343 | 286772469 | 379.2345 | – 0.55 |
| $a_{4SF}$ – $H_2O$ | $C_{18}H_{31}N_4O_3$ | 351.2392 | 28604150 | 351.2396 | – 1.11 |
| $b_{3PL}$, $b_{3PF}$ | $C_{19}H_{26}N_3O_3$ | 344.1968 | 44758821 | 344.1974 | – 1.92 |
| $a_{3PL}$, $a_{3PF}$ | $C_{18}H_{26}N_3O_2$ | 316.2018 | 4040034 | 316.2025 | – 2.63 |
| $b_{3SF}$ – $H_2O$ | $C_{14}H_{22}N_3O_3$ | 280.1650 | 20303286 | 280.1661 | – 4.04 |

**Amanexitide (1):** colorless needles (MeOH), mp 210 °C (decomp.); $[\alpha]_D^{26}$ – 104.5 (*c* 0.2, MeOH); positive ESIMS *m/z* 1072 $[M + K]^+$, 1056 $[M + Na]^+$, 1034 $[M + H]^+$; negative ESIMS *m/z* 1068 $[M + Cl]^-$, 1032 $[M \quad H]^-$; positive ESIMS[2] see Figures 1 and 2; FTICRMS see Table 1 and Figure 3; HRESIMS *m/z* 1034.5715 $[M + H]^+$ (calcd. for $C_{56}H_{75}N_9O_{10}$, 1034.5715).

**ESIMS/MS Analysis.** A bench-top triple quadrupole mass spectrometer model API 2000 (MDS Sciex, Concorde, Canada) equipped with a Sciex TurboIonSpray probe was used. Compound **1** in MeOH was directly infused into the mass spectrometer (flow-rate = 10 $\mu$L/min) to acquire the full-scan and product ion mass spectra. The Q1 full scan spectrum of **1** was first conducted to obtain its corresponding protonated molecular ion at *m/z* 1034.5. The product ion scan spectrum was further acquired by transmitting the protonated molecular ion via Q1 and scanning for products resulting from fragmentation in the collision cell. The final electrospray conditions were as follows: nitrogen curtain gas, 40 L/min; ion-spray voltage 4800 V, source block temperature 120 °C, desolvation gas temperature 400 °C. Nitrogen was used as drying gas and nebulising gas at flow rates of approximately 50 and 450 L/h. The low-energy collision-activated dissociation (CAD) experiment was performed using nitrogen as collision gas, and a collision energy of 40 eV was used. Significant fragment ions are labeled with the four-part descriptor ($x_{nJZ}$) proposed by Ngoka and Gross.[15]

**FTICRMS Analysis.** FTICR-MS analysis was performed on a commercial APEX Qe FT-MS instrument equipped with a 9.4 T superconducting magnet and a Combi ESI/MALDI ion source (Bruker Daltonics, Billerica MA) using electrospray ionization. Compound **1** (1 mg) was dissolved in 1 mL of MeOH and 1 $\mu$L of this solution was diluted in 1 mL of 0.1% formic acid and 80% MeCN afterward. The flow rate was 1 mL/min and the temperature of dry gas (nitrogen) was set to

120 °C. The Q front-end consists of a quadrupole mass filter followed by a hexapole collision cell. By switching the potentials on the exit lenses appropriately under the control of the data acquisition computer, ions could be accumulated either in the hexapole of the Combi ESI source, or in the hexapole collision cell of the Q front-end, prior to transfer to the FTMS analyzer cell. Mass spectrum was obtained by accumulating ions in the collision hexapole and running the quadrupole mass filter in nonmass-selective (RF-only) mode so that ions of a broad *m/z* range (200–1200) were passed to the FTMS analyzer cell. The accumulation time in collision cell was set at 0.5 s, the cell was opened for 4500 ms, 16 experiments were collected for one spectrum. The instrument was externally calibrated using quadruple-, triple- and double- charged ions of angiotensin I. It results in typical mass accuracy below 1 ppm. After the analysis the spectra were apodized using *sin* apodization with one zero fill.

**Amino Acid Analysis.** Compound **1** (1 mg) in 1 ml of 6 N HCl containing 4% thioglycolic acid were heated at 110 °C for 24 h in a sealed tube. After cooling, the solution was concentrated to dryness. The hydrolysate was dissolved in anhydrous solution of 3 N HCl in 2-propanol and heated at 110 °C for 30 min. The reagent were evaporated under reduce pressure. The residue was dissolved in $CH_2Cl_2$ (0.5 ml) and 0.5 ml trifluoracetic anhydride was added. The mixture was kept in a screw-capped tube at 110 °C for 20 min. The reagents were evaporated and the mixture was subjected to GC-MS analysis using a Shimadzu GCMS-QP2000Plus apparatus, equipped with a Chirasil-l-Val (*N*-propionyl-l-valine-tertbutylamide polysiloxane) quartz capillary column. The carrier gas was nitrogen (1.1 bar). Column temperature was initially 50 °C and gradually increased to 130 °C at 3 °C/min, then increased to 190 °C at 10 °C/min. For GC-MS detection, an electron ionization system was used with an ionization energy of 70 eV. Five amino acid derivatives were detected from the mixture. By analysis of EIMS data, four of them were identified to be the 2-propyl esters of *N*-trifluoroacetylated Val ($t_R$ = 12.0), Leu ($t_R$ = 13.9), Pro ($t_R$ = 16.9), and Phe ($t_R$ = 22.8), and one was *N*,*O*-ditrifluoroacetylserine 2-propyl ester ($t_R$ = 14.7). Next, these derivatives were prepared from standard amino acids. The derivatives from hydrolysate were then re-analyzed and their retention times were compared with those of the derivatives from standard amino acids. The amino acid residues in **1** were confirmed and all were determined to have the L configuration.

## Acknowledgments

We thank Prof. Tai-Hui Li, Guangdong Institute of Microbiology, Guangzhou, China for the authentication of plant material. This work was supported by the Knowledge Innovation Program of the Chinese Academy of Sciences (No. KSCX2-YW-N-036).

**Open Access** This article is distributed under the terms of the Creative Commons Attribution License which permits any use, distribution, and reproduction in any medium, provided the original author(s) and source are credited.
